# 肺癌微创手术的生物学意义之思考

**DOI:** 10.3779/j.issn.1009-3419.2018.03.09

**Published:** 2018-03-20

**Authors:** 清泉 罗, 佳 黄

**Affiliations:** 200030 上海，上海交通大学附属胸科医院，上海市肺部肿瘤临床医学中心 Shanghai Lung Tumor Clinical Medical Center, Shanghai Chest Hospital, Shanghai Jiaotong University, Shanghai 200030, China

**Keywords:** 微创外科手术, 肺肿瘤, 生物学微创, Minimal invasive surgery, Lung neoplasms, Biological minimal invasiveness

## Abstract

能够尽量缩短手术时长、加速术后快速康复的微创手术才能达到生物学意义上的微创。肺癌微创手术有多种手术方式，包括单孔胸腔镜手术和多孔胸腔镜手术，其中多孔胸腔镜手术又包含达芬奇机器人手术。外科手术机器人作为目前微创技术领域的前沿，已广泛应用于各个外科领域。本手术组多孔机器人肺手术时长平均为（91.51±30.80）min，相较单孔手术时长大大缩短。目前机器人手术系统尚存在一些缺陷，也缺少全国统一的机器人胸部手术诊疗规范，有待未来技术发展和大型前瞻性随机对照试验来解决。

## Abstract (en)

Minimal invasive surgery with short operation time and enhanced recovery after surgery can truly achieve biological minimal invasiveness. The minimal invasive lung cancer surgery includes several kinds, such as uniportal video-assisted thoracoscopic surgery (VATS) and multi-portal VATS. Robotic-assisted thoracic surgery (RATS) can be categorized into multi-portal VATS. As a frontier technology of minimal invasive surgical technique, surgical robotic system has been broadly applied in many areas. The average RATS operation time is (91.51±30.80) min among our team, which is much shorter than reported uni-portal VATS operation time. For now, RATS has some drawbacks and is lacking of national practice guidelines, which, we believe, will be solved by technology development and largescale randomized controlled trials.

现代医学起初从纯生物学角度研究宿主、环境和病因三大因素的动态平衡。但随着人类社会发展和疾病谱的变化，人们逐渐认识到原有医学模式的不足，提出了生物-心理-社会医学模式。而微创手术从起初单纯缩小体表创伤的理念进步到如今涵盖减少体表创伤-缩短手术和麻醉时间-加速术后康复-降低免疫打击-提高生活质量-保证心理健康等数个方面的多元化的生物学意义上的整体微创。

除了致力于病灶的安全彻底切除外，外科医生还应从整体的角度来看待手术。手术会对患者造成全身和长期的影响，包括免疫系统，消化系统甚至生活质量、心理健康等，这些也是创伤，如何将这些创伤影响降低到最小，是现代外科医生应该关注的问题，如此才能做到真正的微创。

目前治疗肺癌的微创手术主要包括单孔胸腔镜手术和多孔胸腔镜手术。由于目前达芬奇机器人手术仍为多孔，故也归类于多孔胸腔镜手术。尽管并无标准的单孔电视胸腔镜手术(video-assisted thoracoscopic surgery, VATS)的定义，单孔VATS手术概念包括单孔切口(3 cm-5 cm)、软性胸撑撑开主操作孔、全内镜下操作。单孔VATS肺叶除定义为使用单孔切口，不撑开肋骨并在完全腔镜下实施的解剖性肺叶或肺段切除和系统性淋巴结清扫术。1998年，单孔VATS由意大利学者Migliore等率先开展。2004年，Rocco等率先提出单孔VATS的概念。2013年，Roc等率先报道了600例肺楔形切除、活检等简单操作为主的单孔VATS。2011年，Gonzalez等率先报道了单孔全腔镜肺切除术，2013年报道了解剖性肺切除的102例。此后，Gonzalez团队又率先开展了单孔VATS全肺切除、单孔VATS肺动脉袖式切除、单孔VATS支气管袖式切除、单孔VATS解剖肺段切除、单孔VATS纵隔肿瘤切除、单孔VATS气管及隆凸切除术等。Hsu等报道了首例多中心单孔VATS解剖性肺切除治疗肺癌的研究。Wang等报道了单孔VATS的围手术期各项指标与多孔VATS手术相仿。

但是就手术时长而言，单孔手术的时间明显长于多孔手术。Xie等报道了截至2016年最大宗的单孔解剖性肺切除手术的单中心回顾性研究，文献中表示单孔腔镜肺手术的平均手术时间为(135.0±31.0) min。罗清泉等发表了单中心700例机器人辅助胸腔镜解剖性肺切除手术的回顾性研究，其中表明多孔机器人肺手术时长平均为(91.51±30.80) min，相较单孔手术其时间大大缩短，减少患者的全麻时间及麻醉药物的使用，减少术中器械游离时对正常组织的伤害，将内部脏器的损伤降至最低，最大程度保留肺功能，提高手术安全性，达到生物学意义上的微创。单孔微创和多孔微创手术均为肺微创手术中可选择的手术入径，外科医生应根据自身习惯、患者病情或其他特殊情况选择不同的手术入径，而不应拘泥于其中一种，所有的选择均应基于患者的安全及肿瘤彻底切除的原则之上。

目前市场上唯一商用的可用于外科领域的机器人手术系统是达芬奇机器人手术系统(Intuitive Surgical, Sunnyvale, CA)，该系统由4个部分组成：①机械手臂系统；②外科医生控制台；③高分辨率的3D双眼内视系统；④有着7种自由度且在机械臂长轴方向上有2个自由度的内转腕系统。主刀外科医生坐在与手术台有一定距离的控制台对机械手臂进行操纵，而手术台和患者则与有着3条-4条机械手臂的床旁机械臂系统极为贴近^[1, 2]^。自2002年Mel等^[3]^报道了第一例机器人辅助肺叶切除手术后，机器人在胸外科领域得到逐步推广。根据美国卫生保健质量和研究署数据库，2013年机器人肺叶切除手术在所有肺叶切除手术中占比达到11%，相较2009年的1%有显著提高。2011年-2015年末，全球开展机器人肺叶切除手术的中心数量增长90%，同期机器人肺叶切除手术数量增加了185%^[4]^。中国大陆2016年一年共完成机器人胸外手术2, 119例，同比去年增长38.5%，2009年-2016年末，中国大陆共完成机器人胸外手术4, 737例。

由于机器人外科手术系统模拟了人手操作与人眼成像，所以与普通胸腔镜相比，机器人手术系统具有独特的意向运动能力、活动范围甚至可大于人手的灵活的内转腕机械手臂。外科医生可真正实现将自己的手"送入"患者体内，有别于传统腔镜的"筷子式"操作。此外，机器人手术系统还特有"滤过系统"，可过滤术者手部细微颤抖。这些特点大大提高手术流畅度和术者的舒适程度，达到更精准的骨骼化结构、解剖式切除，明显缩短手术时间、减少麻醉药用量。由于机械手臂孔均较小，无需明显撬动肋骨，且使用保护性trocar，对肋间神经影响较小，患者术后疼痛相较开放手术和普通腔镜明显改善^[5, 6]^，进而加速患者术后快速康复。目前达芬奇机器人系统(Si, Xi)均可支持单孔操作，进一步减少患者体表创伤。

目前的国内外相关文献均表明机器人肺手术是安全且高效的^[7]^。从患者角度而言，机械手臂孔体表直径8 mm-10 mm，肋间神经压迫少，术后疼痛较传统胸腔镜和开放手术程度轻、恢复快，术后住院时间明显缩短，伤口更为美观，术后心理健康及长期生活质量可能更优。从术者角度出发，手术舒适性提高，无需长时间站立，无需频繁刷手消毒，且术野更为立体清晰。

此外，机器人内转腕系统使其在辅助外科医生清扫淋巴结时具有独特优势，有相关文献表明机器人清扫淋巴结效果优于普通胸腔镜和开放手术。目前由上海市胸科医院肺部肿瘤临床医学中心外科牵头发起的多中心前瞻性机器人与开胸手术对比的随机对照临床研究正在进行中，希望对将来机器人肺部手术规范制定有所帮助。

早期肺癌治愈率较高，应追求对早期肺癌患者做到生物学意义上的整体微创。而对于某些进展期的肺癌患者而言，机器人手术系统可以以较小的体表创伤为其完成较复杂的手术，如支气管袖式切除、支气管动脉双袖切除等。作为外科领域最新技术代表的机器人手术系统将精准医疗和生物学意义的整体微创完美结合在一起。

目前机器人外科手术系统存在的主要缺陷一是缺乏触觉反馈，二是其高昂的费用。期望在未来的机器人手术系统中能够通过改进设备及软件方面的升级，使术者在操控台操作时能够具有综合的、实时的、持续的力反馈，还期望能够具有更高的精确运动缩放功能，以便于更精准的操作。2017年5月，威高集团发布"妙手S"机器人手术系统，目前已成功为6例患者实施手术，相信在不久的将来便会对现有的机器人手术系统的市场价格形成挑战。同时也需要大量前瞻性随机对照研究进一步证明机器人外科手术系统的有效性和安全性，未来仍需建立全国统一的机器人胸部手术诊疗规范。
